# Associations of sleep characteristics with alpha‐synuclein in cerebrospinal fluid in older adults

**DOI:** 10.1002/acn3.51204

**Published:** 2020-09-19

**Authors:** Xiao‐Tong Wang, Feng‐Tao Liu, Yan‐Lin Bi, Xue‐Ning Shen, Wei Xu, Jian Wang, Lan Tan, Jin‐Tai Yu

**Affiliations:** ^1^ Department of Neurology Qingdao Municipal Hospital Qingdao University Qingdao China; ^2^ Department of Neurology and Institute of Neurology Huashan Hospital Shanghai Medical College Fudan University Shanghai China; ^3^ Department of Anesthesiology Qingdao Municipal Hospital Qingdao University China

## Abstract

**Objective:**

Sleep disorders as a preclinical symptom of synucleinopathies become more prevalent in older adults. Synucleinopathies might be caused by the abnormal aggregation of alpha‐synuclein in the brain, which was indicated by alpha‐synuclein levels in cerebrospinal fluid (CSF). We aimed to investigate associations of sleep characteristics with CSF alpha‐synuclein in older adults.

**Methods:**

Our study recruited 536 cognitively intact individuals (aged between 40 and 90 years old) from the Chinese Alzheimer’s Biomarker and Lifestyle study. Sleep behaviors were assessed by Pittsburgh Sleep Quality Index and total alpha‐synuclein in CSF was measured by enzyme‐linked immune‐sorbent assay. We used multiple linear and non‐linear regression models for research.

**Results:**

Significant non‐linear associations of CSF alpha‐synuclein with sleep time and duration were revealed. Individuals who went to bed and fell asleep too early or late tended to have lower CSF alpha‐synuclein (reflection point for time to bed and fall asleep were 10:26 p.m. and 10:40 p.m.). Lower CSF alpha‐synuclein was also observed in individuals with either excessive or insufficient sleep duration (reflection point: 7.24 hours). Besides, overall poor sleep quality (β = −0.0621; *P* = 0.0242), longer sleep latency (β = −0.0415; *P* = 0.0174) and lower sleep efficiency (β = 0.0036; *P* = 0.0017) showed linear associations with lower CSF alpha‐synuclein. Sleep disturbances and daytime dysfunction were not significantly associated with CSF alpha‐synuclein.

**Interpretation:**

Poor sleep was associated with lower levels of CSF alpha‐synuclein in older adults, which may provide new insight into the prevention of synucleinopathies.

## Introduction

Disturbed sleep becomes more prevalent with growing age.^1^ Various sleep problems, especially rapid eye movement (REM) and sleep behavior disorder (RBD), have been reported as prodromal symptoms in many neurodegenerative disorders like Parkinson’s disease (PD), multiple system atrophy (MSA) and dementia with Lewy bodies (DLB).^2^ All these diseases share a similar progression of Lewy neurite and Lewy body (LB) pathology mainly caused by the abnormal aggregation of alpha‐synuclein (α‐syn) in the brain, which thus are termed as alpha‐synucleinopathies.^3^ A study showed that sleep disorders might be associated with the deterioration of brainstem nuclei,^4^ where LB pathology might occur.^5^, ^6^ Accumulation of LBs might begin before symptoms appear^7^, ^8^, ^9^ and LBs were also observed in healthy individuals.^10^ Other previous studies also reported that the glymphatic system might promote clearance of accumulated proteins in the brain when sleeping.^11^, ^12^, ^13^, ^14^

Sleep disorders might play an important role in neurodegenerative diseases. However, the influence of sleep disorders on cerebrospinal fluid (CSF) α‐syn levels which indirectly reflect synuclein accumulation in the brain^15^ has not been well investigated. Therefore, in the present study, we examined the associations of sleep characteristics with α‐syn in CSF among cognitively intact older adults and we hypothesize that poor sleep behaviors are associated with lower CSF α‐syn levels.

## Methods

### CABLE study

Participants were recruited from the Chinese Alzheimer’s Biomarker and Lifestyle (CABLE) study.^16^ CABLE has been an ongoing study majorly focused on risk factors and biomarkers of neurodegenerative disorders since 2017. Individuals were Han Chinese recruited at Qingdao Municipal Hospital, Shandong Province, China. Demographic information and medical history were collected via a structured questionnaire and an electronic medical record system. People with genetic diseases (or family history of genetic diseases), central nervous system infection, major neurological diseases, psychological disorders, such as anxiety and depression assessed by neuropsychiatric inventory (NPI), and other severe systemic diseases were excluded. All participants underwent clinical and neuropsychological assessment, biochemical testing, as well as blood and CSF sample collection. Participants were aged between 40 and 90 years and the cognitive diagnoses were based on the criteria made by National Institute on Aging and the Alzheimer's Association (NIA‐AA).^17^, ^18^ CABLE was approved by the institutional review board of Qingdao Municipal Hospital and written informed consent was obtained from all participants or their guardians according to the Declaration of Helsinki.

### Study participants

Our cross‐sectional study included 536 cognitively intact participants from CABLE who failed to meet the criteria for cognitive impairment. Participants finished the major sleep questionnaire, as well as the CSF sample collection. General cognitive function was assessed by Chinese‐Modified Mini‐Mental State Examination (CM‐MMSE).^19^ Possible scores of this scale range from 0 to 30 with higher scores representing better cognitive performance. These tests were administered by specially trained neurologists. Complete information of each participant was also available from the CABLE cohort, including age, gender, years of education, CM‐MMSE, time points of CSF sampling, as well as self‐reported history of alcohol intake, type 2 diabetes, and hypertension.

### Sleep characteristics

The Pittsburgh Sleep Quality Index (PSQI)^20^ used in the CABLE has been completed by included individuals the day before lumbar puncture. The PSQI is a self‐reported questionnaire evaluating sleep quality and disturbances during the last month, including subjective sleep quality, sleep latency (minutes to fall asleep), sleep duration (hours), habitual sleep efficiency, sleep disturbances, use of sleeping medication, and daytime dysfunction. Each question is rated on a four‐point severity scale ranging from 0 to 3 and the global score of the questionnaire ranges from 0 to 21. The questionnaire also included some specific questions, such as “When do you go to bed and get up?” and “How long do you fall asleep?” Data on self‐reported sleep quality and medication use for sleep disorders the day before lumbar puncture were only used as supplementary data, as approximately one‐fifth of the included participants did not provide these data.

### CSF total α‐syn

CSF was sampled by lumbar puncture from participants in 15ml polypropylene tubes before being sent to the local lab within 2 hours. CSF samples were centrifuged at 2000*g* for 10 minutes. Then the CSF samples were transferred into 200ul polypropylene tubes before being stored in the fridge at −80°C. CSF total α‐syn was measured using a pre‐coated plate with an enzyme‐linked immunosorbent assay (ELISA) kit (Legend Max™ Human α‐Synuclein, BioLegend Corporation) on the EnSpire Multimode Plate Reader (PerkinElmer Corporation). All CSF samples were measured in duplicate in each run by experienced operators who were blinded to demographic information and medical history. CSF α‐syn concentration measured using standard curves with 4‐parameter curve fitting is considered to be accurate. The within‐batch precision value was 6.24% for α‐syn. The freeze‐thaw cycle may not be conducted more than two times, as previous investigations have shown the stability of CSF biomarkers may decrease after more than two freeze‐thaw cycles.^21^, ^22^ Strict quality control measures were needed to minimize the preanalytical and analytical variabilities by controlling the coefficient of variation (CV) under 20 and the possible influence of hemolysis on α‐syn values. Moreover, in order to avoid subjective factors, we also had separate researchers for information collection and CSF measurement.

### Data analysis

The basic characteristics of the participants from CABLE were summarized using descriptive statistics. Individuals whose concentration of total α‐syn in CSF > 4000 pg/mL (which greatly exceeded the 95% confidence limit for the range of all α‐syn data^23^) were excluded as outliers (total = 11) to ensure the stability of the results. Due to the skewed distribution of CSF α‐syn levels, the transformation was then performed to approximate a normal distribution via the “car” package of R software, which may facilitate comparisons between modalities. In order to avoid the influence of individual differences, possible confounding factors, including age, sex, education years, CM‐MMSE scores, time points of CSF sampling, as well as self‐reported history of alcohol intake, type 2 diabetes and hypertension (yes or no), were adjusted for.

Multiple linear regression models were run separately for the associations of each sleep condition indicator and normalized α‐syn levels in CSF, using α‐syn as a dependent variable and sleep characteristics as independent variables. Samples were divided into different severity groups of PSQI indicators. A group with a few individuals (n < 10) was combined with its adjacent group into a new group in order to avoid unreliable results. We also examined interactions between each possible confounding variable and α‐syn levels in CSF in order to explore whether there were strata effects. If any potential interaction (*P* < 0.1) appears, subgroup analysis will be further performed. In order to investigate if there was also a nonlinear (such as U‐shaped) association between sleep dysfunction and CSF α‐syn levels, non‐linear regression analyses via the quadratic model (y = αx^2^ + βx + c) were employed. Significant non‐linear associations were observed when the coefficients of the quadratic term were significantly larger (U‐shaped) or smaller (reverse U‐shaped) than zero. When the same sleep item had significance in both linear and non‐linear models, we chose the one with greater R‐squared which implied higher goodness of fit.

Statistical significance was set at a two‐tailed *P* < 0.05. Bonferroni method was further applied to all sleep characteristics for multiple corrections. We explored the associations between α‐syn levels in CSF and sleep behaviors using R version 3.5.1 (R Project for Statistical Computing; http://www.r‐project.org) and GraphPad Prism version 8.0 statistical software.

## Results

### Characteristics of included participants in CABLE

The demographic data of the included participants are shown in Table 1. A total of 536 individuals were included from CABLE. They were cognitively unimpaired (mean CM‐MMSE score = 27.31) individuals in the late midlife with a mean age of 62.84 years old (standardized deviation [SD]: 10.83 years old) and a female proportion of roughly 42.54%. The mean total score of PSQI was 4.71. The median concentration of CSF total α‐syn was 1271.11pg/ml, ranging from 485.9 to 3957.33pg/ml. Further details are also shown in Table 1.

**Table 1:** Basic characteristics of included participants.

Variable	No. (n = 536)
Age (y, mean ± SD)	62.84 ± 10.83
Sex (female, %)	228 (42.54%)
Education (years, mean ± SD)	9.72 ± 4.30
CM‐MMSE scores (mean ± SD)	27.31 ± 3.06
History of alcohol intake (yes, %)	164 (30.60%)
History of type 2 diabetes (yes, %)	92 (17.16%)
History of hypertension (yes,%)	217 (40.49%)
Clock time to bed (earliest to latest)	05:00 p.m. to 02:00 a.m.
Clock time to fall asleep (earliest to latest)	05:30 p.m. to 03:30 a.m.
Clock time to get up (earliest to latest)	02:00 a.m. to 10:30 p.m.
Time in bed (hours, mean ± SD)	8.03 ± 1.21
Specific sleep latency (minutes, mean ± SD)	24.28 ± 25.45
Specific sleep duration (hours, mean ± SD)	6.67 ± 1.42
Specific sleep efficiency (%, mean ± SD)	83.66 ± 15.46
The score of sleep quality in PSQI (0/1/2/3)	174/253/88/21
The score of sleep latency in PSQI (0/1/2/3)	212/176/80/68
The score of sleep duration in PSQI (0/1/2/3)	184/139/170/43
The score of sleep efficiency in PSQI (0/1/2/3)	309/107/46/74
The score of sleep disturbance in PSQI (0/1/2/3)	231/299/5/1
The score of sleep medication use in PSQI (0/1/2/3)	492/11/10/23
The score of daytime sleepiness in PSQI (0/1/2/3)	505/20/4/7
Total score of PSQI (mean ± SD)	4.71 ± 3.56
Self‐reported sleep quality the day before lumbar puncture (good/fair/bad/NA)	232/135/64/105
Medication use the day before lumbar puncture (yes/no/NA)	41/370/125
Time points of CSF sampling (earliest to latest)	8:30 a.m. to 9:10 p.m.
CSF α‐syn levels (pg/ml)	
median	1,271.11
(min, max)	(485.90, 3,957.33)

SD, standardized deviation; CM‐MMSE, Chinese‐Modified Mini‐mental State Examination; NA, not accessible; PSQI, Pittsburgh Sleep Quality Index; CSF, cerebrospinal fluid; α‐syn, alpha‐synuclein; min, minimum; max, maximum.

### Non‐linear associations of sleep characteristics with CSF α‐syn levels

**Figure 1:**
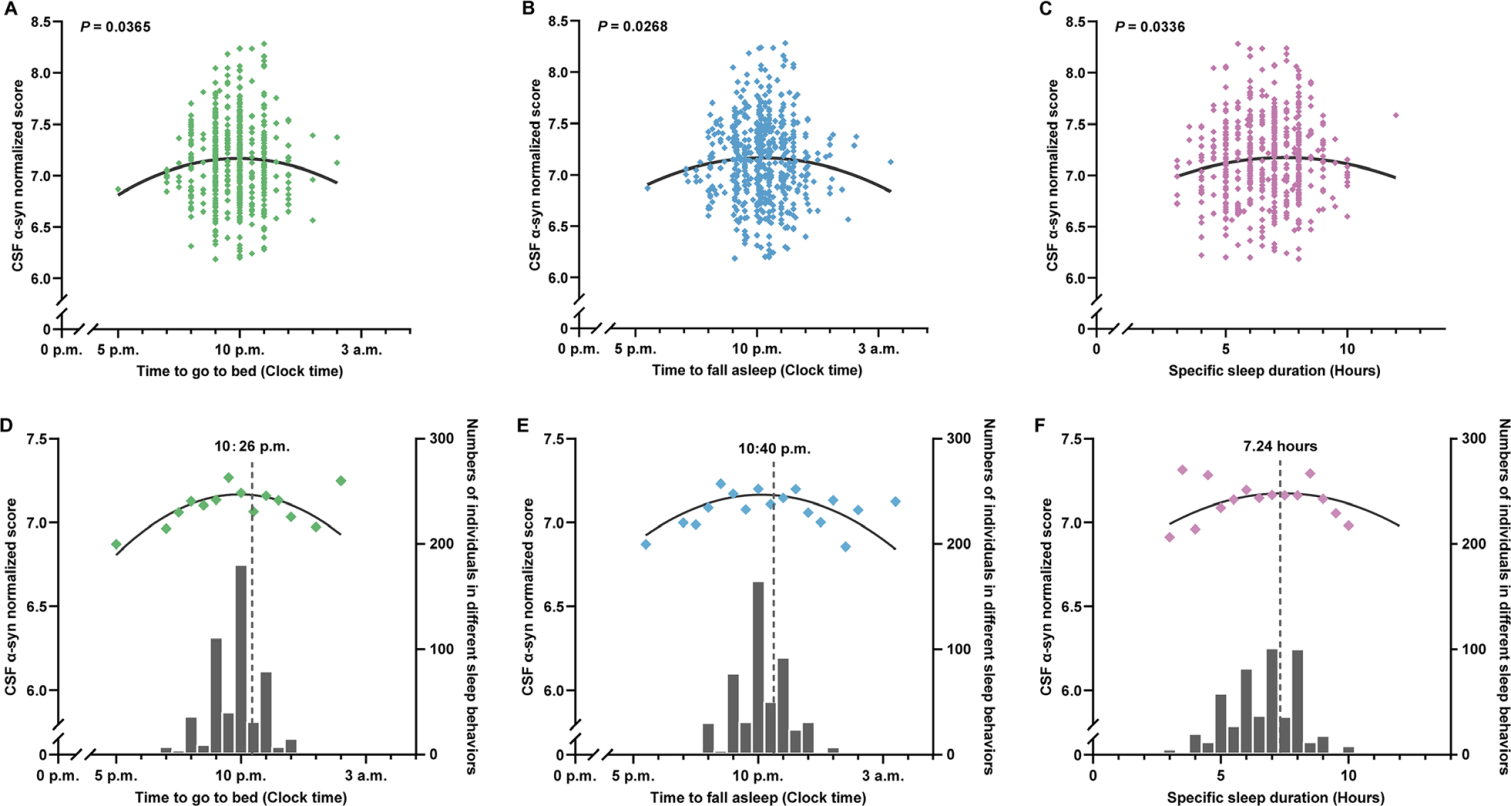
Non‐linear associations of sleep time and duration with CSF α‐syn levels. Time to go to bed (1A) and time to fall asleep (1B) showed reverse *U*‐shaped associations with lower CSF α‐syn. Either insufficient or excessive sleep duration is also associated with lower α‐syn levels in CSF (1C). Mean CSF α‐syn levels (normalized) of each time point to sleep are observed to have a reverse U‐shaped association with the corresponding numbers of individuals in every sleep time point shown in the bar graph (1D). Similar non‐linear relationship is shown between the time to fall asleep and the mean normalized levels of CSF α‐syn at each time point (1E). Numbers of individuals corresponding to each time point are shown in the bar graph. The average CSF α‐syn levels (normalized) of each sleep hour showed a non‐linear trend (1F), suggesting either insufficient or excessive sleep duration associated with lower CSF α‐syn levels. The numbers of individuals with each sleep hour are shown in the bar graph.

**Figure 2:**
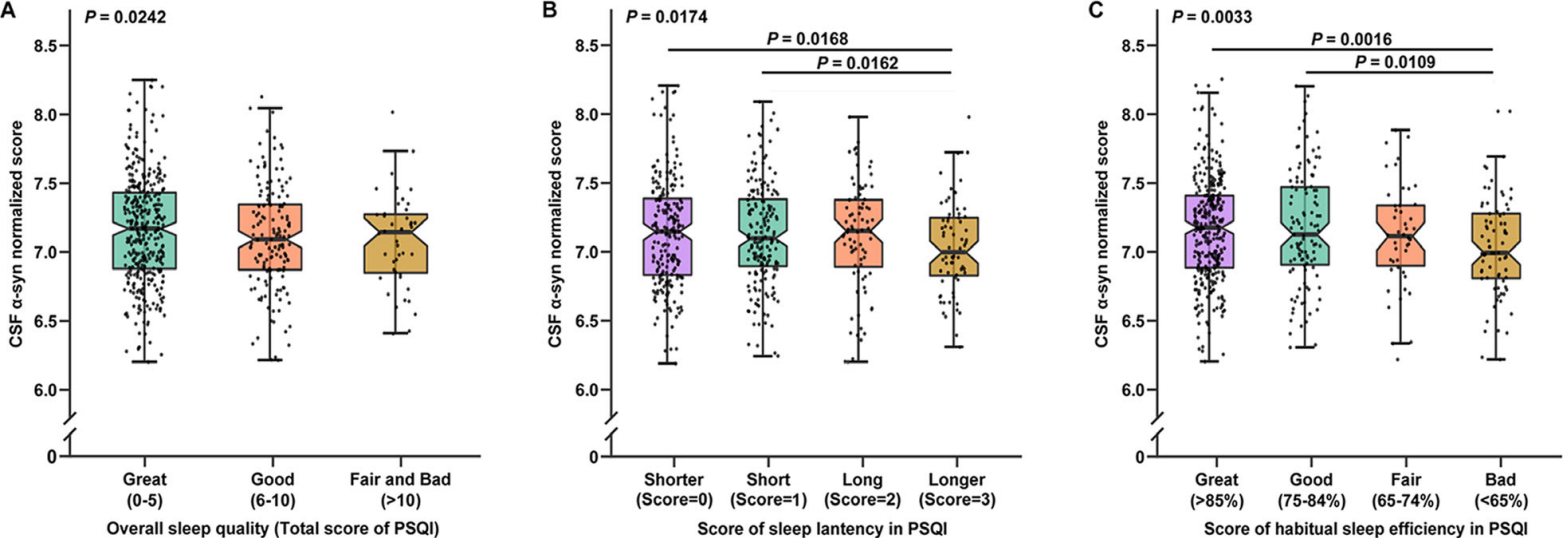
Linear associations of global sleep quality and score of characteristics in PSQI with CSF α‐syn. Poor sleep quality is significantly associated with lower α‐syn levels in CSF (2A). The score of sleep latency has been revealed to have a significant relationship with CSF α‐syn levels (2B), where a higher score is associated lower CSF α‐syn levels. A higher score of sleep efficiency indicating lower sleep efficiency is also associated with lower CSF α‐syn levels (2C).

Non‐linear associations between specific sleep characteristics and CSF α‐syn levels are shown in Table 2. Significant trends of reverse U‐shaped associations of α‐syn levels in CSF with sleep time including time to go to bed (*P* = 0.0365) and time to fall asleep (*P* = 0.0268) (calculated via time to bed and specific latency) are revealed in Figure 1A and B. Moreover, a similar significant association between CSF α‐syn levels and specific sleep duration (*P* = 0.0336) is also revealed in Figure 1C. In addition to the specific distribution, the mean CSF α‐syn levels (normalized) for all sleep time points and durations instead of all the scattered points might show clearer non‐linear trends in Figure 2, where the number of individuals in corresponding sleep time points and durations were shown via the bar graph. The recommended time to bed and time to fall asleep was approximately 10:30 p.m. and 10:40 p.m., where maximum points (‐β/[2α]) (Table 2) indicated the fitted curve reached the peak (Figure 1D and E). Both insufficient and excessive sleep durations were associated with lower levels of CSF biomarkers. The reflection point might be 7.24 hours for the highest α‐syn level in CSF (Figure 1F), which may suggest that the proper sleep duration is approximately 7 to 7.5 hours a day. Apart from detailed information on sleep, a score reflecting medication use in PSQI (*P* = 0.0193) also showed a significant non‐linear association with α‐syn levels in CSF, while differences in sample size need to be taken into the consideration (Supplementary Table S1). Non‐linear associations between scores reflecting other aspects in PSQI and CSF α‐syn levels are also shown in Table S1.

**Table 2:** Non‐linear associations between specific sleep characteristics and CSF α‐syn levels.

Specific sleep characteristics	α coefficients^†^	β coefficients^†^	Extreme point^&^	*P* values^†^
Clock time to go to bed	‐0.017132	0.357644	10:26 p.m.	**0.0365**
Clock time to fall asleep	‐0.015855	0.337972	10:40 p.m.	**0.0268**
Clock time to get up	0.000834	‐0.028122	04:52 p.m.	0.9256
Time in bed (hours)	0.003121	‐0.079709	12.77	0.6149
Specific sleep latency (minutes)	0.000025	‐0.003917	78.34	0.0598
Specific sleep duration (hours)	‐0.013372	0.193586	7.24	**0.0336**
Specific sleep efficiency (%)	‐0.000041	0.009889	120.60	0.5206

CSF, cerebrospinal fluid; α‐syn: alpha‐synuclein; α: coefficient of the quadratic term; β: coefficient of the primary term; &: maximum or minimum points (‐β/[2α]) indicates where fitted curve reached the peak or bottom; †: adjusted for age, sex, education years, CM‐MMSE, time points of sampling, self‐reported history of type 2 diabetes, hypertension and alcohol intake; bold text: adjusted *P* < 0.05 and considered as statistical significance.

### Linear associations of sleep disorders with CSF α‐syn levels

**Figure 3:**
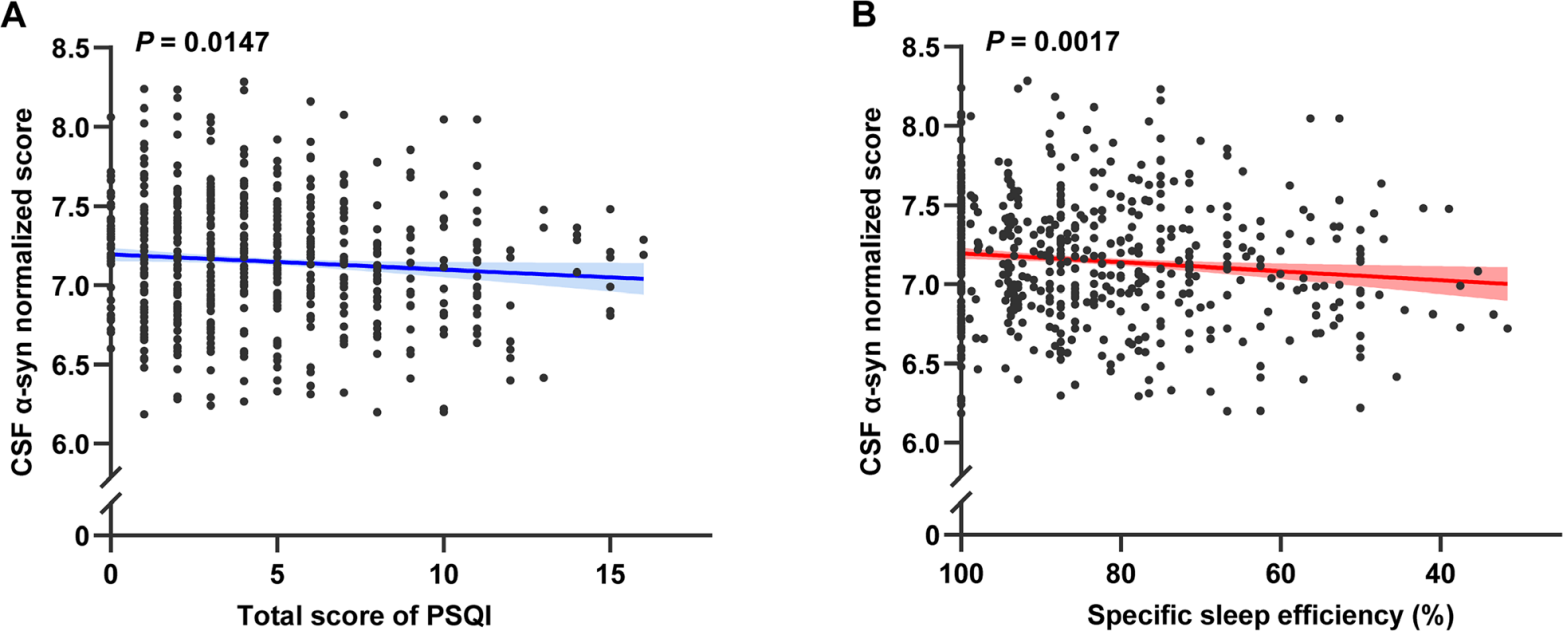
Linear associations of PSQI total score and sleep efficiency with CSF α‐syn. A higher total score of PSQI indicating poor sleep quality is associated with lower CSF α‐syn levels (3A). Lower sleep efficiency is also strongly associated with lower CSF α‐syn levels (3B).

In addition to the non‐linear associations, some significant linear associations between sleep characteristics and CSF α‐syn levels were also reported. The overall sleep quality (β = −0.0621; *P* = 0.0242) (Figure 2A) determined by the total score of PSQI has shown a significant association with CSF α‐syn levels. To be specific, individuals with higher scores indicating poorer sleep quality had lower α‐syn concentrations in CSF. The score of sleep latency in PSQI (β = −0.0415; *P* = 0.0174) was inversely correlated with CSF α‐syn levels in Figure 2B. Moreover, a lower score of habitual sleep efficiency in PSQI (β = −0.0479; *P* = 0.0033) was observed to have a stronger association with higher CSF α‐syn levels in Figure 2C. Besides, higher total score of PSQI (β = −0.0122; *P* = 0.0147) and lower specific sleep efficiency (β = 0.0036; *P* = 0.0017) also had significant associations with lower CSF α‐syn levels, which are shown in Figure 3A and B, respectively. Moreover, the association between specific sleep efficiency and CSF α‐syn was also survived Bonferroni correction (*P* < 0.0031). Sleep efficiency might be a component of overall sleep quality. Therefore, CSF α‐syn levels decreased with worsening sleep quality, indicating that sleep quality may be linked to synucleinopathies. No interaction effect of confounding factors was shown in our study. Further details of linear regression results are shown in Table S2.

## Discussion

In our study, unsuitable sleep time points and insufficient or excessive sleep duration may contribute to decreased CSF α‐syn levels. Moreover, poor sleep quality was also associated with a decrease in CSF α‐syn levels. All these findings might indicate that poor sleep might be associated with lower CSF α‐syn levels in elders without neurological disorders, suggesting a possible role of sleep improvement in the further disease‐modifying therapies for neurodegenerative disorders.

α‐Syn is a normal constituent of the enteric nervous system in each individual.^24^, ^25^ CSF α‐syn levels were likely to reflect the levels of soluble α‐syn proteins in the brain regions which might be affected by the aggregation of these proteins and formation of LBs.^15^ Abnormal accumulated α‐syn in the brain might lead to neurodegenerative diseases, which may need to be cleared to maintain homeostasis. Waves of CSF flow during sleep have been reported to benefit protein aggregate clearance,^14^ possibly via the glymphatic system and the exchange interstitial fluid (ISF) and CSF.^13^ The process of the glymphatic system function is marked by the doubling of the CSF clearance rate, alongside a 60% increase in the interstitial space in the brain during sleep time compared to the wake time.^11^, ^12^ Moreover, the circadian cycle has been reported to control the clearance of protein aggregates^26^ and modulate the accumulation of α‐syn.^27^, ^28^ Therefore, poor sleep quality or unsuitable sleep time points might interfere with the clearance of abnormally accumulated α‐syn in the brain, which may lead to a decrease in CSF total α‐syn levels.

In our research, we found sleep time points and sleep duration were significantly associated with CSF α‐syn levels in cognitively intact individuals. To be specific, individuals with unsuitable sleep time points showed relatively lower CSF α‐syn levels compared with those with a suitable time to sleep. A cross‐sectional analysis showed lower CSF total α‐syn levels in the individuals with PD^29^ and a longitudinal study of nurse shift workers also reported an increased risk of PD compared to nurses without night shift jobs,^30^ both of which indicated that disturbed sleep might lead to lower CSF α‐syn levels. Wake‐up time in our study showed negative nonlinear association with CSF α‐syn, which might need further investigation, as sleep‐wake rhythm disturbances were reported associated with the spread of α‐syn aggregation.^27^ Nonlinear associations identified here should be interpreted with caution, as it was also possible that the recommended bedtime might reflect the physiological fluctuation of CSF α‐syn levels and might be influenced by multiple factors in different cohorts, for example, geographical position.^31^ Besides, either excessive or insufficient sleep duration might not be recommendable. Several studies reported that excessive sleep duration might increase the risk of PD and DLB,^32^, ^33^ and some other studies found that insufficient sleep duration was likely to increase the risk of parkinsonism or some motor symptoms.^34^, ^35^ Therefore, insufficient or excessive sleep duration would possibly lead to lower CSF α‐syn levels and even the development of neurodegenerative diseases, which was consistent with our results. The U‐shaped association of sleep duration and CSF α‐syn levels might be explained from the perspective of sleep quality. Previous studies reported individuals with insufficient and excessive sleep duration often experienced poor sleep quality,^36^, ^37^ which might also contribute to the lower CSF α‐syn. Supporting for this hypothesis, in our study, poor sleep quality were significantly associated with lower CSF α‐syn.

Overall sleep quality is the general quality of some specific sleep dimensions, such as sleep latency and sleep efficiency. The effects of decreased sleep quality might aggravate the neurodegenerative process in PD.^27^ More directly, poorer sleep quality was likely to contribute to neurodegeneration, which could be indicated by CSF α‐syn levels. Difficulty initiating sleep (a manifestation of insomnia) occurred more frequently in individuals with synucleinopathies compared with matched controls.^38^, ^39^ Sleep efficiency, the percentage of time spent asleep in bed, has also been observed to decrease in individuals with synucleinopathies.^38^, ^39^, ^40^ Other specific sleep dimensions, such as sleep disturbances, medication taking, and daytime sleepiness, though showed non‐significant associations with CSF α‐syn in our study, were reported associated with α‐syn propagation in the nervous system,^40^, ^41^ which might also lead to lower levels of CSF α‐syn. The inference that sleep disorders may often company low CSF α‐syn levels could possibly be applied not only in the patients with synucleinopathies but also in cognitively intact individuals, as sleep disorders usually appeared before the diagnosis of synucleinopathies.^2^ This may explain why the poor sleep group of cognitively intact individuals included in our study showed lower CSF α‐syn levels.

Additional longitudinal evidence was also necessary to elucidate whether sleep disorders were associated with the changes in CSF α‐syn levels during the follow‐up. Epidemiological studies suggested that disturbed sleep may increase the risk of developing PD,^40^ which was confirmed by a large population‐based study in individuals without pre‐existing PD.^42^ A 3‐year longitudinal study found isolated RBD participants had decreased CSF α‐syn levels, whereas slightly higher CSF α‐syn levels appeared in healthy individuals.^23^ Hence, sleep disorders may contribute to lower CSF α‐syn levels and finally lead to neurodegeneration. Individuals with synucleinopathies often have longer latencies,^7^, ^8^, ^9^ and sleep behaviors as potential clinical symptoms could be easily detected by the patients or their guardians before motor symptoms appear. Therefore, sleep monitoring and management may provide potential value for disease prediction.

Limitations of our study also should be mentioned. Individuals from CABLE had no follow‐up records. In addition, questionnaires used for assessing sleep status were all self‐reported; conclusions were based on observational studies; and there was a lack of objective measures like polysomnography system for sleep monitoring. All these might lead to a relative lack of objectivity in our results. Future studies could enrich assessment methods to reach a more reliable conclusion or even find out new associations between sleep behaviors and CSF α‐syn.

## Conclusions

Poor sleep quality such as longer sleep latency or lower efficiency, as well as insufficient or excessive sleep duration and unsuitable sleep time, were associated with lower CSF α‐syn levels in healthy older adults. Sleep monitoring and management are expected to provide new insights into the diagnosis and prevention of synucleinopathies.

## Authors' Contributions

JTY and LT conceptualized and designed the study. XTW, FTL, YLB, and WX conducted the study. XTW, FTL, WX, XNS, and JTY analyzed and extracted data. XTW, FTL, LT, JW, and JTY wrote the first draft of the manuscript. All authors reviewed the manuscript.

## Conflict of Interests

None authors have financial disclosures and conflicts of interest.

## Supporting information


**Table S1.** Non‐linear associations between scores of sleep characteristics in PSQI and CSF α‐syn levels.
**Table S2.** Linear associations between sleep characteristics and CSF α‐syn levels.
