# Supplementary material for: Associations of sleep characteristics with alpha‐synuclein in cerebrospinal fluid in older adults
Source: Ann Clin Transl Neurol. 2020 Sep 19;7(10):2026–34. doi: 10.1002/acn3.51204 (PMC7545588; doi:10.1002/acn3.51204)
Supplement: Supplementary file 1 — Table S1. Non‐linear associations between scores of sleep characteristics in PSQI and CSF α‐syn levels. Table S2. Linear associations between sleep characteristics and CSF α‐syn levels. [file ACN3-7-2026-s001.docx]

| **Table S1. Non-linear associations between scores of sleep characteristics in PSQI and CSF α-syn levels** | | | | |
| --- | --- | --- | --- | --- |
| **Sleep characteristics in PSQI** | **α coefficients** | **β coefficients** | **Extreme point^&^** (scores) | **P values^†^** |
| Subjective sleep quality | -0.006016 | -0.021860 | 1.8168 | 0.7879 |
| Sleep latency | -0.021135 | 0.016045 | 0.3796 | 0.2485 |
| Sleep duration | -0.025093 | 0.044358 | 0.8839 | 0.2209 |
| Habitual sleep efficiency | -0.025180 | 0.021396 | 0.4249 | 0.2167 |
| Sleep disturbances | 0.110274 | -0.150904 | 0.6842 | 0.0515 |
| Use of sleeping medication | 0.115418 | -0.348255 | 1.5087 | **0.0193** |
| Daytime dysfunction | 0.045170 | -0.087480 | 0.9683 | 0.3664 |
| Total score of PSQI^*^ | 0.000202 | -0.014709 | 36.408 | 0.8594 |

PSQI: Pittsburgh Sleep Quality Index; CSF: cerebrospinal fluid; α-syn: alpha-synuclein; α: coefficient of quadratic term; β: coefficient of primary term; &: maximum or minimum points (-β/[2α]) indicates where the fitted curve reached the peak or bottom; †: adjusted for age, sex, education years, CM-MMSE, time points of sampling, self-reported history of type 2 diabetes, hypertension and alcohol intake; bold text: adjusted *P* < 0.05 and considered as statistical significance

| **Table S2. Linear associations between sleep characteristics and CSF α-syn levels** | | | | | |
| --- | --- | --- | --- | --- | --- |
| **Sleep characteristics** | **β coefficients^#^** | ***P* values^#^** | **β coefficients^†^** | ***P* values^†^** |  |
| Clock time to go to bed | 0.005119 | 0.746 | 0.021232 | 0.2160 |  |
| Clock time to fall asleep | -0.002299 | 0.877 | 0.008581 | 0.5916 |  |
| Clock time to get up | -0.021390 | 0.197 | -0.018051 | 0.2956 |  |
| Time in bed (hr) | -0.019220 | 0.176 | -0.027941 | 0.0563 |  |
| Specific sleep latency (min) | -0.000841 | 0.212 | -0.001127 | 0.1064 |  |
| Specific sleep duration (hr) | 0.015430 | 0.202 | 0.016630 | 0.1816 |  |
| Specific sleep efficiency (%) | 0.002835 | 0.0104 | 0.003602 | **0.0017**^*^ |  |
| The score of subjective sleep quality in PSQI | -0.024070 | 0.262 | -0.035900 | 0.1045 |  |
| The score of sleep latency in PSQI | -0.030820 | 0.0648 | -0.041541 | **0.0174** |  |
| The score of sleep duration in PSQI | -0.016410 | 0.347 | -0.018768 | 0.2980 |  |
| The score of habitual sleep efficiency in PSQI | -0.038010 | 0.0163 | -0.047929 | **0.0033** |  |
| The score of sleep disturbances in PSQI | -0.018360 | 0.576 | -0.018079 | 0.5944 |  |
| The score of use of sleeping medication in PSQI | -0.023270 | 0.364 | -0.027372 | 0.3030 |  |
| The score of daytime dysfunction in PSQI | 0.011230 | 0.783 | 0.023455 | 0.5717 |  |
| Total score of PSQI | -0.009608 | 0.046 | -0.012179 | **0.0147** |  |
| Global sleep qualtiy in PSQI | -0.045800 | 0.0867 | -0.062140 | **0.0242** |  |

CSF: cerebrospinal fluid; α-syn: alpha-synuclein; PSQI: Pittsburgh Sleep Quality Index; β: coefficient of primary term; #: unadjusted model; †: adjusted for age, sex, education years CM-MMSE, time points of sampling, self-reported history of type 2 diabetes, hypertension and alcohol intake; bold text: adjusted *P* < 0.05 and considered as statistical significance; *: still statistical significance after Bonferroni correction (adjusted *P* < 0.0031)
